# High HIV prevalence and associated factors in Lesotho: Results from a population-based survey

**DOI:** 10.1371/journal.pone.0271431

**Published:** 2022-07-28

**Authors:** Amee Schwitters, Stephen McCracken, Koen Frederix, Reese Tierney, Masebeo Koto, Nahima Ahmed, Kyaw Thin, Trudy Dobbs, Sakhile Sithole, Mosilinyane Letsie, Bharat Parekh, Hetal Patel, Sehin Birhanu, Lubbe Wiesner, Andrea Low

**Affiliations:** 1 Centers for Disease Control and Prevention, Maseru, Lesotho; 2 Division of Global HIV and TB, US Centers for Disease Control and Prevention, Center for Global Health, Atlanta, GA, United States of America; 3 ICAP in Lesotho, Mailman School of Public Health, Columbia University, Maseru, Lesotho; 4 School of Public Health at Georgia State University, Atlanta, GA, United States of America; 5 Lesotho Ministry of Health, Maseru, Lesotho; 6 ICAP at Columbia University, Mailman School of Public Health, Columbia University, New York, NY, United States of America; 7 Division of Clinical Pharmacology, Department of Medicine, University of Cape Town, Cape Town, South Africa; Clinton Health Access Initiative, SOUTH AFRICA

## Abstract

Despite extensive global efforts, sub-Saharan Africa remains disproportionately affected by the HIV epidemic. This generalized epidemic can be seen in Lesotho which in 2014 the HIV prevalence rate of those aged 15–49 years was 24.6%, with and incidence of 1.9 new infections per 100-person-year exposures. To better understand the impact of Lesotho’s national HIV response and significant predictors associated with HIV infection, the Lesotho Population-based HIV Impact Assessment was conducted. This survey provided a nationally representative sample of individuals aged 15–59 years old in which participants were tested for HIV and given an individual questionnaire that included socio-demographic and behavioral risk questions. The association of factors between survey questions and HIV incident was assessed using logistic regression. Multivariate logistic regression models for men and women were constructed for each outcome using variables known to be or plausibly associated with recent or chronic infection. Overall annualized incidence among people aged 15–49 was 1.19% (95% CI 0.73–1.65) per year. The overall prevalence of HIV was 25.6% with women having significantly higher prevalence. Multiple variables, including decreased wealth status, lower education levels, marital status, condom use at first sex, and circumcision (men only) were identified as being significantly associated with HIV infection for both men and women. In combination with improving the awareness of HIV status, an increased focus is needed on AGYW and men 35–49 years old to prevent new infections. HIV education and prevention programs should focus heavily on younger age groups prior to and soon after sexual debut to prevent HIV transmission. The findings of the survey showed significant room for improvement in increasing awareness of HIV status and reinforcing the need for continued HIV prevention and treatment efforts in Lesotho to prevent new infections.

## Introduction

Despite extensive global efforts, sub-Saharan Africa remains disproportionately affected by the HIV epidemic. Approximately 25.5 million HIV-positive persons, or 70% of the global number of HIV-positive persons, live in the region [1]. While rates of HIV testing and awareness have increased, substantial gaps remain within specific populations [2]. In prior studies, men have been found to test less frequently than women and report higher levels of anticipated stigma [3, 4]. AIDS-related stigma leads to barriers in access to HIV prevention, testing, and care for individuals [5]. Stigma remains perpetuated at both the individual and structural levels and has been shown to negatively impact both individual- and community-level HIV testing [6, 7]. Data from recent national surveys show that while approximately 77% of diagnosed persons are on treatment, there are approximately 40% of persons who are HIV-positive and unaware of their status [2]], and therefore not on virally suppressive antiretrovirals (ART). Evidence shows that viral suppression successfully reduces or eliminates the risk of HIV transmission to sexual partners and newborns and is a key component of epidemic control [8].

Lesotho, a mountainous country surrounded by the Republic of South Africa, has a population of approximately 2 million with 66% of the population residing in rural areas [9]. Lesotho has a generalized HIV epidemic [10]. In 2014, prevalence of HIV infection among persons aged 15–49 years was 24.6%, with an incidence of 1.9 new infections per 100 person-years of exposure, and low ART coverage (42%) [11–13]. In April 2016, Lesotho became the first country in sub-Saharan Africa to adopt the World Health Organization (WHO) recommendations for universal initiation of ART for all HIV-positive persons, regardless of CD4 count (i.e. “Test and Start”), with nationwide implementation occurring in June 2016 [14, 15].

The Lesotho Population-based HIV Impact Assessment (LePHIA) was a national household-based survey conducted across all ten districts in Lesotho between November 2016 and May 2017. LePHIA was conducted to understand the impact of Lesotho’s national HIV response. Using both individual and community level variables, this analysis aims to describe the various predictors of HIV infection among individuals aged 15–49 years living in Lesotho; including the potential impact of stigma on new infections among men.

## Methods

### Survey design and participants

LePHIA utilized a two-stage sampling design to select a nationally representative sample of individuals aged 15–59 years in 418 out of the 5,390 enumeration areas (EAs) across the ten districts of Lesotho, as previously described [16]. Within each district, EAs were selected based on probability proportional to each size; the size of an EA is defined by the number of households in it at the time of the 2016 census. Within districts, the urban/rural distribution of selected EAs were proportional to their distribution in the census. Within the EAs, a random, systematic sample of households was selected at rates designed to yield a self-weighting (i.e. equal probability) sample within each district to the extent feasible. Among included households, after giving informed consent, head of households completed a household questionnaire that included a roster of all household members who resided in or had slept in the household the previous night. Eligible household members were then asked to provide consent to participate in an individual questionnaire that included socio-demographic and behavioral risk questions and to home-based rapid HIV testing. A guardian or parent provided permission for interviewers to approach individuals aged 15–17 years who were then asked for assent for all procedures. Written informed consent was documented at each stage via electronic signature. The LePHIA protocol and data collection tools were approved by the Lesotho Research and Ethics Committee, and the Institutional Review Boards at Columbia University Medical Center and the US Centers for Disease Control and Prevention (CDC).

### Procedures

Survey staff used Google Nexus 9 tablets to administer the questionnaire to participants during face-to-face interviews in a private area. The questionnaire included questions on lifetime and recent sexual behaviors. A migration module captured mobility outside Lesotho (defined as having lived outside Lesotho for more than one month continuously).

Venipuncture was performed by trained nurses, and rapid HIV testing was conducted using Determine HIV-1/2 Rapid Test (Alere, Japan [Abbott, USA]) and was confirmed with the Uni-Gold HIV Test (Trinity Biotech, Ireland). Capillary blood draws were used in cases of failed venous blood draws. Counselling was provided, with referral to a preferred facility for all those who tested seropositive. Laboratory verification of all HIV-positive results was done using the Geenius HIV 1/2 supplemental assay (Bio-Rad, USA). HIV-1 RNA in plasma and dried blood spots was measured using real-time polymerase chain reaction (Cobas Taqman, Roche, USA). Recent HIV infection was determined using the HIV-1 Limited Antigen (LAg) Avidity Enzyme Immunoassay (Sedia Biosciences, USA) for HIV-positive specimens. Samples with a normalized optical density less than 1.5, that were not virally suppressed (defined as HIV RNA <1000 copies/ml for LePHIA) with assay performance characteristics of a mean duration of recent infection = 130 days (95% CI: 118–142), a time cutoff (T) = 1.0 year and percentage false recent = 0.00 were classified as a recent infection [16]. Only those persons testing positive for HIV were tested for recency and viral load status. Antiretroviral (ARV) analytes for the three most commonly prescribed antiretrovirals (lopinavir, nevirapine, and efavirenz) with long half-lives were tested on HIV-positive DBS using high-resolution liquid chromatography (HRLC) at the University of Cape Town in South Africa [17]. The time from ingestion to detectability is 1.5–2.5 days for lopinavir, 8–9 days for nevirapine, and 12–28 days for efavirenz [18].

### Statistical analysis

Design weights were calculated based on sampling design, including probability of household selection, adjusted for non-response at the household, individual and biomarker levels using the SI-CHAID software ® (Statistical Innovations, USA). Post-stratification weights were calculated to reflect the age distribution of the 2016 Lesotho census ] using a ratio adjustment factor that included variables for gender and age. Analyses were done in Stata version 15.1 using probability weights and jackknife replicates for variance estimation. All reported proportions are weighted, unless otherwise indicated. Incidence estimates were based on the number of HIV infections identified as recent with the HIV-1 LAg Avidity plus VL algorithm and were obtained using the formula recommended by the WHO Incidence Working Group and Consortium for Evaluation and Performance of Incidence Assays [19]. Community level variables were generated as a weighted mean value across the EA, with viremia defined as having a viral load >1000 copies/ml, and the denominator including all adults, regardless of serostatus. The association between factors included in the survey questionnaire and HIV incidence for persons 15–49 or prevalence for persons 15–59 was assessed using logistic regression. Multivariate logistic regression models for men and women were constructed for each outcome using variables known to be or plausibly associated with chronic infection. Given the relatively high rate of misreporting and HIV negative status in those later shown to be on ARVs in LePHIA [16]], we also analyzed predictors of this misreporting, to assess how stigma might play an ongoing role in the epidemic in Lesotho.

## Results

### Participation

Of the 10892 selected households, 9403 and 8824 were occupied and interviewed, respectively. The overall household unweighted response rate (RR) was 93.2% (91.2% and 91.4% in urban and peri-urban areas, respectively, and 94.9% in rural areas). There were 11682 adults with interview and HIV test results (6892 women [RR 87.3%] and 4790 men [RR 78.1%]) (Table 1). The median age of adult participants was 30 years (interquartile range [IQR], 22–41). More than half (58.3%) resided in rural areas or reported that they had been tested for HIV in the 12 months prior to the survey (56.1%). Forty-eight percent of participants were married or living with a partner and 59.7% of women and 50.9% of men had attended secondary school or higher. More men (30.5%) than women (18.0%) reported having lived outside Lesotho (Table 2).

**Table 1:** Interview and blood draw response rates amongst participants aged 15–59 years, Lesotho Population-based HIV Impact Assessment 2016–2017.

	Total	
Result	Male %	Female %
Eligible Individuals, ages 15–24 years		
Number of eligible individuals	2,159	2,708
Interview response rate (unweighted)	86.9	93.9
Interview response rate (weighted)	86.3	93.8
Blood draw response rate (unweighted)	90.1	92.7
Blood draw response rate (weighted)	89.4	92.3
Eligible Individuals, ages 15–49 years		
Number of eligible individuals	5,473	6,871
Interview response rate (unweighted)	86.8	95.1
Interview response rate (weighted)	86.2	94.9
Blood draw response rate (unweighted)	88.4	91.6
Blood draw response rate (weighted)	87.8	91.0
Eligible Individuals, ages 15–59 years		
Number of eligible individuals	6,135	7,893
Interview response rate (unweighted)	87.4	95.4
Interview response rate (weighted)	86.8	95.1
Blood draw response rate (unweighted)	88.8	91.9
Blood draw response rate (weighted)	88.2	91.3

**Table 2:** Demographic characteristics of participants aged 15–59 years (N = 12877; men, 5361; women, 7526), Lesotho Population-based HIV Impact Assessment 2016–2017*.

Characteristic	Men %, (n)	Women %, (n)	Total %, (n)	Characteristic	Men %, (n)	Women %, (n)	Total %, (n)
Age (years)				Wealth quintile			
15–19	17.8 (919)	17.3 (1155)	17.5 (2047)	Lowest	18 (996)	16.7 (1337)	17.3 (2333)
20–24	16.5 (767)	16.7 (1199)	16.6 (1966)	Med-Low	19 (965)	17.6 (1327)	18.3 (2292)
25–29	16.0 (702)	15.6 (1054)	15.8 (1756)	Medium	19.5 (920)	20 (1380)	19.7 (2300)
30–34	14.6 (609)	13.5 (865)	14.1 (1465)	Med-High	20.8 (936)	21.2 (1397)	21 (2333)
35–39	11.4 (573)	10.4 (769)	10.9 (1342)	Highest	22.7 (925)	24.6 (1471)	23.7 (2396)
40–44	8.1 (427)	8.0 (625)	8.1 (1052)				
45–49	6.1 (352)	6.4 (474)	6.2 (826)	Schooling attended, years			
50–54	5.3 (285)	6.4 (490)	5.8 (775)	No education	8.5 (461)	1.4 (108)	5 (569)
55–59	4.3 (324)	5.7 (500)	5.0 (824)	Primary	40.6 (2052)	38.9 (2867)	39.8 (4919)
				Secondary	40.3 (1840)	48.9 (3328)	44.6 (5168)
Area of residence				Post-secondary	10.5 (389)	10.8(609)	10.6 (998)
Urban	47 (2029)	50.7 (3180)	48.9(5209)				
Rural	53 (2713)	49.3(3732)	51.1 (6445)	Marital status			
				Married/living with partner	44.1 (2019)	51.9 (3606)	48 (5625)
District				Single	46.7 (2243)	30.6 (2034)	38.6 (4277)
Berea	14.1 (688)	14.4 (985)	14.3 (1673)	Divorced/Separated/Widowed	9.2 (480)	17.5 (1272)	13.4 (1752)
Botha-Bothe	5.6 (361)	5.1 (456)	5.3 (817)				
Leribe	17.6 (886)	17.1 (1242)	17.4 (2128)	Currently attending school			
Mafeteng	8.2 (528)	8.6 (735)	8.4 (1263)	No	78.8 (3726)	77.9 (5455)	78.3 (9181)
Maseru	32.7 (1430)	31.3 (1907)	32.0 (3337)	Yes	21.2 (1016)	22.1 (1457)	21.7 (2473)
Mohale’s Hoek	5.5 (376)	6.0 (587)	5.7 (963)				
Mokhotlong	4.6 (289)	4.8 (427)	4.7 (716)	Work in the last 12 months for which cash or goods were received			
Qacha’s Nek	2.4 (200)	2.7 (309)	2.5 (509)	No	54.9 (2779)	66.9 (4781)	60.9 (7560)
Quthing	3.7 (275)	4.3 (415)	4.0 (690)	Yes	45.1 (1963)	33.1 (2131)	39.1 (4094)
Thaba Tseka	5.6 (328)	5.8 (463)	5.7 (791)				
				First sex before age 15			
Sex partner last 12 months				No	86.9 (4116)	95.4 (6585)	91.2 (10701)
None	9.9 (501)	13.9 (1014)	11.9 (1515)	Yes	13.1 (626)	4.6 (327)	8.8 (953)
1	48.8 (2299)	63 (4374)	55.9 (6673)				
2	14.6 (672)	7.4 (510)	11 (1182)	Ever lived outside Lesotho			
3+	11.7 (518)	2 (123)	6.8 (641)	No	69.4 (3310)	82.2 (5689)	75.8 (8999)
Refused./Don’t know	1.9 (84)	1.6 (107)	1.7 (191)	Yes	30.6 (1432)	17.8 (1223)	24.2 (2655)
Never	13.1 (668)	12 (784)	12.5 (1452)				
				Tested for HIV, last 12 months			
				Yes	50.5 (2707)	61.7 (4644)	56.1 (7224)
				No	49.5 (2654)	38.3 (2882)	43.9 (5653)

*Percentages are survey weighted. Note that totals might not add to 100% due to rounding. Not all categories total overall numbers due to missing responses within the category. Participants must have responded “yes” to ever-attended school prior to being asked about current attendance. Work was limited to employment for which compensation was received. Overall number total in Table 1 is representative of the unweighted interview response weight totals and includes persons who did not receive a blood draw.

Overall, 37.6% of households were directly affected by HIV (at least one HIV-positive member), with 80.5% of households with one HIV-positive member, 17.7% with two HIV-positive members, and 1.8% had three or more HIV-positive members.

### Incidence

**Fig 1:**
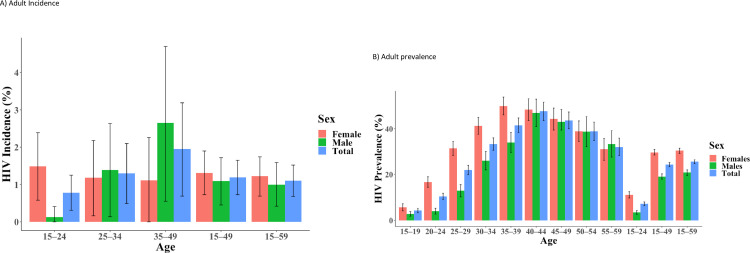
HIV incidence and prevalence in adults aged 15–59 years by age and sex in Lesotho, 2016–2017. A) Adult incidence B) Adult prevalence.

Overall annualized incidence among people aged 15–49 was 1.19% (95% CI 0.73–1.65) per year; 1.31% (95% CI 0.73–1.90) among women and 1.09% (95% CI 0.45–1.72, Fig 1A) among men. This corresponds to approximately 10000 new cases of HIV each year. Incidence was highest among men aged 35–49 years (2.65%, 95% CI 0.55–4.70), and lowest among men aged 15–24 (0.13%, 95% CI 0–0.41%). Among women, incidence ranged from 1.18% (95% CI 0.16–2.18) among those aged 25–34 years, to 1.49% (95% CI 0.58–2.39) among those aged 15–24 years (Fig 1A).

### Prevalence

Overall prevalence was 25.6% (95% CI 24.7–26.4), corresponding to approximately 306,000 persons aged 15–59 years living with HIV. Women had significantly higher prevalence (30.4%; 95% CI 29.2–31.5) than men (20.8%; 95% CI 19.6–22.0). Prevalence ranged from 2.8% (95% CI 1.8–3.8) among men aged 15–19 years to 46.9% (95% CI 40.9–52.9) among men aged 40–44 years. Prevalence ranged from 5.7% (95% CI: 4.1–7.2) among women aged 15–19 years to 49.9% (95% CI 46.0–53.8) among women aged 35–39 years (Fig 1B).

Among men, each one year increase in age (from age 15 years was associated with an increased odds of HIV infection (aOR: 1.29; 95% CI:1.23–1.35; p-value: <0.001). Men who lived in an urban setting had higher odds of being infected with HIV compared to those who lived in a rural setting (aOR: 0.63; 95% CI:0.47–0.83; p-value: 0.002) as well as those living in the mountain region (aOR: 0.70; 95% CI:0.54–0.91; p-value:0.010). The odds of being HIV-positive decreased for both men and women as the individuals education level increased (aOR: 0.95; 95% CI:0.66–1.36; p-value: <0.001). Men who were single were less likely to be HIV-positive (aOR: 0.63; 95% CI:0.48–0.84; p-value: <0.001) than men who were married or living with a partner. Men who were divorced, separated, or widowed (aOR: 2.47; 95% CI:1.59–2.66; p-value: <0.001) had higher odds of being HIV-positive than men who were married or living with a partner. Men who reported more than three sexual partners in a lifetime had higher odds of being HIV-positive than men reporting one sexual partner in his lifetime (aOR: 1.59; 95% CI:1.08–2.32; p-value: 0.02). Men who had a medical circumcision had lower odds (aOR: 0.55; 95% CI:0.44–0.70; p-value: <0.001) of being HIV-positive compared to men who had a traditional circumcision. Men who used a condom during their first sexual experience had lower odds (aOR: 0.73; 95% CI:0.57–0.93; p-value: 0.012) of being HIV-positive compared to those who did not use a condom in their first sexual experience (Table 3).

**Table 3:** Predictors of HIV infection (prevalence) in participants aged 15–59 years by sex, Lesotho Population-based HIV Impact Assessment 2016–2017*.

	Men				Women			
	Odds Ratio (OR) (95% Confidence Interval [CI])	p-value	Adjusted Odds Ratio (aOR) (95% CI)	p-value	OR (95% CI)	p-value	aOR (95% CI)	p-value
**Per additional year of age (15–49)**	1.32 (1.27–1.37)	<0.001	1.29 (1.23–1.35)	<0.001	1.26 (1.23–1.29)	<0.001	1.22 (1.19–1.26)	<0.001
**Area of Residence**								
Urban	Ref.	---	Ref.	---	Ref.	---	Ref.	---
Rural	0·94 (0·81–1·11)	0·46	0·63 (0·47–0·83)	0·002	0·94 (0·84–1·05)	0.24	0.83 (0.70–0.98)	0·026
**Ecological Zone**								
Lowlands	Ref.	---	Ref.	---	Ref.	---	Ref.	---
Foothills	0.97 (0.75–1.26)	0.84	0.95 (0.66–1.36)	0.751	0.86 (0.72–1.04)	0.115	0.79 (0.62–1.01)	0.055
Mountains	1.00 (0.84–1.19)	0.999	0.70 (0.54–0.91)	0.010	0.90 (0.79–1.04)	0.151	0.73 (0.60–0.88)	0.002
**Wealth Quintile** (per one unit increase)	0.89 (0.84–0.94)	<0.001	0.91 (0.81–1.03)	0.004	0.95 (0.92–0.98)	0.003	0.89 (0.83–0.96)	<0.001
**Years of Schooling**								
Per additional educational level attended	0.88 (0.60–0.90)	<0.001	0.95 (0.66–1.36)	0.002	0.88 (0.86–0.90)	<0.001	0.91 (0.89–0.94)	<0.001
**Marital Status**								
Married/living Together	Ref.	---	Ref.	---	Ref.	---	Ref.	---
Single	0·19 (0·15–0.23)	<0·001	0.63 (0.48–0.84)	<0·001	0.49 (0.43–0.56)	<0·001	1.32 (1.09–1.61)	0.007
Divorced/Sep./Widowed	2·47 (1·96–3·11)	<0·001	2.05 (1.59–2.66)	<0·001	3·18 (2.77–3.66)	<0·001	2.41 (2.06–2.83)	<0·001
**Lived Outside of Lesotho**								
Never	Ref.	---	Ref.	---	Ref.	---	Ref.	---
Once	1·74 (1·41–2·14)	<0·001	1.06 (0.83–1.34)	0.65	1.58 (1·35–1·85)	<0·001	1.18 (0.98–1.43)	0.08
Twice	1.75 (1.22–2.50)	0.004	1.00 (0.66–1.53)	0.98	2.06 (1.42–2.99)	<0.001	1.43 (0.96–2.12)	0.07
Three or More	1.91 (1.49–2.45)	<0.001	1.05 (0.81–1.36)	0.72	1.49 (1.16–1.91)	<0.001	1.02 (0.77–1.37)	0.88
**Age at First Sex**								
Younger than 15 years	0·56 (0·44–0·72)	<0·001	1.03 (0.77–1.39)	0.73	1.44 (1.17–1.92)	0.019	1.36 (1.00–1.86)	0.05
Older than 15 years	1.59 (1.15–2.19)	0.007	0.93 (0.59–1.45)	0.84	1.61 (1.15–2.27)	0.008	1.08 (0.68–1.74)	0.730
**Use of Condom at First Sex**								
No	Ref.	---	Ref.	---	Ref.	---	Ref.	---
Yes	0.29 (0.24–0.36)	<0.001	0.73 (0.57–0.93)	0.01	0.43 (0.38–0.49)	<0.001	0.70 (0.59–0.83)	<0.001
**Overall Number of Partners**								
1	Ref.	---	Ref.	---	Ref.	---	Ref.	---
2	1.09 (0.70–1.72)	0.68	1.22 (0.75–2.00)	0.41	1.85 (1.56–2.21)	<0.001	1.79 (1.50–2.15)	<0.001
3	1.19 (0.79–1.78)	0.39	1.21 (0.79–1.87)	0.36	2.59 (2.21–3.03)	<0.001	2.40 (2.04–2.83)	<0.001
4	2.00 (1.28–2.89)	0.001	1.59 (1.08–2.32)	0.02	3.68 (3.09–4.40)	<0.001	3.10 (2.53–3.80)	<0.001
5	1.55 (1.08–2.22)	0.019	1.88 (1.22–2.90)	0.006	0.89 (0.72–1.09)	0.24	2.43 (1.78–3.36)	<0.001
**Male Circumcision**								
Traditional/Uncircumcised	Ref.	---	Ref.	---	---	----	---	---
Medical Circumcision	0.36 (0.30–0.43)	<0.001	0.55 (0.44–0.70)	<0.001	---	---	---	---

*Data are survey weighted. Multivariable models constructed using variable chosen *a priori* looking at known risk factors for HIV from relevant published literature.

The odds of a woman being HIV-positive increased with each year of age (beginning at age 15) (aOR:1.22; 95% CI:1.19–1.26; p-value: <0.001) and peaked at age 44. Amongst women, living in a rural area was associated with a decreased odds of being HIV positive compared to women who lived in an urban setting (aOR: 0.83; 95% CI:0.70–0.98; p-value: 0.026). Women who reported living in the foothills (aOR; 0.79; 95% CI:0.62–1.01; p-value: 0.05) or a mountain region (aOR; 0.73; 95% CI:0.60–0.88; p-value: 0.002) had a decreased odds of being HIV-positive compared to those who lived in the lowlands. Both an increased level of wealth (aOR: 0.89 per unit increase in quintile; 95% CI:0.83–0.96; p-value: 0.004) and education (aOR: 0.91 per additional level attended; 95% CI:0.89–0.94; p-value: <0.001) were significantly associated with a decrease in the odds of a woman being infected with HIV. Women who were single (aOR: 1.32; 95% CI:1.09–1.61; p-value:0.007) or divorced, separated, or widowed (aOR: 2.41; 95% CI:2.06–2.83; p-value: <0.001) had higher odds of being HIV-positive than women who were married or living with a partner. Among women, having a first sexual encounter before the age of 15 years was associated with an increased odds of HIV infection compared to a later sexual debut (aOR: 1.36; 95% CI:1.00–1.86; p-value: 0.05). Women who reported using a condom during their first sexual experience had lower odds of being HIV-positive compared to those who did not (aOR: 0.70; 95% CI:0.59–0.83; p-value:<0.001) as was living outside of Lesotho up to two times (aOR: 1.43; 95% CI:0.96–2.12; p-value: 0.07). Women who had more than one sexual partner in her lifetime had increased odds (aOR: 1.79; 95% CI:1.50–2.15; p-value: <0.001) of being HIV-positive compared to woman who reported one sexual partner (Table 3).

### Awareness and discordance

Despite implementation of Test and Start, 7.6% of individuals participating in LePHIA were aware of their status, but not yet receiving treatment [16]. Self-reported HIV and ART status were checked against ARV metabolites for concordance. Among those who reported that they had not been previously diagnosed, more males (19.1%) had detectable levels of ARVs in their system compared to females (14.0%) and 14.2% of males who denied taking ART had detectable ARVs in their system compared to 11.6% of females [16, 20]. In total, concordance between self-reported HIV-positive status and ARV use and lab-confirmed ARV metabolites was 94.8% [16, 20]. In multivariate analysis, men had a higher odds of discordance between self-reported ARV status and metabolite confirmed ART status. Men reporting individual HIV stigma had a lower odds of discordance if they were married compared with single, divorced or widowed men and lower odds if completed primary or secondary school compared to those without schooling (Table 4).

**Table 4:** Discordance between reported awareness of HIV status and detection of ARVs.

	Women			
Characteristic	OR	p-value	OR	p-value
Age				
15–24	Ref.		Ref.	
25–59	0.29 (0.12–0.73)	0.011	0.44 (0.21–0.89)	0.025
Marital status				
Single/Div./Widowed	Ref.		Ref.	
Currently married	0.51 (0.29–0.89)	0.019	0.60 (0.33–1.11)	0.098
Educational attainment				
None	Ref		Ref	
Primary	0.51 (0.24–1.05)	0.066	0.12 (0.04–0.36)	0.001
Secondary	0.36 (0.15–0.88)	0.027	0.29 (0.09–0.90)	0.03
Tertiary	1.11 (0.28–4.47)	0.873	0.26 (0.04–1.54)	0.13
Wealth quintile	0.90 (0.72–1.13)	0.34	1.12 (0.93–1.35)	0.24
Individual stigma	3.52 (1.34–9.25)	0.013	2.46 (0.84–7.25)	0.098
Community stigma (per 1% increase)	1.02 (1.01–1.04)	0.014	0.98 (0.96–1.00)	0.10
CD4<350	0.88 (0.51–1.53)	0.85	0.96 (0.47–1.95)	0.90

## Discussion

Our results and prior Lesotho Demographic and Health Survey (DHS) data show that HIV prevalence is more or less stable at 25% in Lesotho among persons aged 15–59 years, but increases to almost half of adults aged 40 years or older [11]. Although significant resources have been invested in Lesotho to reduce the number of new infections, HIV incidence remains high and could be a barrier to achieving epidemic control despite progress made with treatment coverage.

We identified key correlates of recent HIV infection. HIV incidence varied by sex and age, with men aged 35 years and older significantly more likely to be recently infected than men aged 15–24. This may be indicative of increased high-risk sexual behavior or new and/or multiple sexual partners as men age.

HIV disproportionately affects women compared to men. This disparity is most evident among young adults, where HIV prevalence among those aged 20–24 years is four times as high among women than among men despite reporting fewer partners and higher condom use [21]. HIV incidence reaches 1.49% among women aged 15–24 years, while new infections among young men are almost zero (0.13%] indicating that women are likely being infected by older male partners, where HIV incidence quickly increases among men 25–49. Prior modelling shows that age-disparate relationships can greatly increase a young woman’s risk of contracting HIV because rates are usually higher in older men than younger men [22, 23]. Research in Zimbabwe showed that a young woman’s risk of contracting HIV increased by approximately 75% if she had a sexual partner 10 or more years older than her [24]. In LePHIA, as reported previously, 11% of women aged 15–19 years or 20–24 years reported having a sexual partner more than 10 years older, with a mean sexual partner age of 25.2 and 28.4 years, respectively [21]. Prior research in South Africa has shown acceptance of pre-exposure prophylaxis (PrEP) among adolescents [25]. Given the potential for age-disparate sexual relationships in Lesotho and the high incidence among adolescent girls and young women future efforts could consider targeting this group with programs such as the implementation of PrEP combined with adherence messages. Testing and prevention messages should be aimed at men 25–49 as incidence begins to significantly increase within this population. Along with implementation of programming, modeling efforts should occur that incorporate difficulties in engaging men in testing and treatment programs with the acceptance of and retention in PrEP among AGYW in Lesotho to see which method is more efficient.

Age is a significant correlate of HIV infection. The HIV prevalence rate in Lesotho among men 25–29 years of age is over three times as high compared to men aged 20–24 years. In our multivariate model, women and men were 1.22 and 1.29 times, respectively, more likely to become infected with HIV for each increased year of age. The increase could be attributed, in part, to women being in their childbearing years during this time because they may be less likely to use condoms or more likely to be sexually active. Age also played a role in early sexual debut and HIV infection status among women. The age at first sexual experience was a significant predictor of HIV positive status as women who had sex for the first time before 15 years were more likely to be HIV positive than women who were older at their first sexual experience. A study conducted among youth in Kenya also found that early sexual debut continues to be a major risk factor for acquiring HIV infection later in life [26]. Adolescents remain an important population to target with HIV prevention messages and condom use. Adolescent women have high rates of HIV positivity in sub-Saharan Africa and key drivers of increased vulnerability include having high-risk, older partners and labor migration [21]. Interventions that can decrease the risk of infection on an individual level include avoiding sex with older partners and delaying sexual debut [22].

Level of education showed a strong correlation with HIV status. For every year of additional schooling completed the odds of becoming HIV-positive significantly decreased. Although primary education in Lesotho is free and compulsory, not all children attend primary school, and fewer children attend and complete secondary school and go on to complete a tertiary education [27]. In LePHIA, 23% of women and 14% of men reported completing only primary school. The relationship between education and HIV risk is complex, but research supports the importance of education in reducing HIV risk [28]. Furthermore, it is important to note that higher levels of educational attainment among women has also been associated with increased control over sexual and reproductive rights [29]. Women who lived in countries with higher rates of complementation of lower secondary school saw a reduce in new HIV infections [29]. Interventions that promote adolescent girl’s education and empowerment in sub-Saharan Africa, such as *Education*, *Plus*, could assist in increased leaves of educational attainment [29].

The risk of HIV differed significantly by marital status among both women and men. In our multivariate model, women who were single, divorced, or widowed were more likely to be infected with HIV compared to women who were married or living with a partner. Men who were single were less likely to be HIV infected compared to married men, while those who were divorced, separated or widowed were more likely to be HIV infected than their married counterparts. This finding of an increased risk among married people is unusual as single people are normally found to be at greater risk. The increased risk associated with marital status has been seen in limited studies in which married women see higher levels of risk due to increased frequency of unprotected sex compared to unmarried women. [30]. As well, specific variables may influence loss of protective factors against HIV transmission, especially among women who marry at an early age as they are more likely to have a lower level of formal education and exposure to HIV prevention programs [31]. The increase in odds for married men could be linked to sexual partnerships outside of marriage in which 29.8% of men in Lesotho reported having extra-marital affairs [17]. Additionally, one out of three married or co-habiting men who reported extra-marital partnerships reported not using a condom [16]. Understanding the determinates of condom use, specifically availability, to create an intervention for men participating in extra-marital partnerships may assist in increasing condom use. A study in Nigeria found that men who believed condoms were both affordable and easy to find were more likely to use condoms thus indicating a need for interventions designed to ensure availability [32]. Along with targeting condom use, focusing on preventing transmission to the married partner may be a feasible approach. As extra-marital partnerships are also a threat to women, a study conducted in Lesotho found that many women who were initially the uninfected partner in a discordant partnership acquired HIV from their husband [10]. An early-initiation of treatment with ARV for prevention of HIV-1 transmission in serodiscordant couples has shown success with a relative reduction of 96% in the number of linked HIV transmissions [33].

The use of a condom at first sexual experience was significant among both men and women in which the odds of HIV infection decreased with condom use. Condom use at first sexual experience is important as correct and consistent condom use is highly effective in preventing the transmission of HIV and other sexually transmitted diseases [34]. Promoting the use of condoms at first sexual experience may also have an impact on a individuals condom use during later sexual experiences. A study in Uganda examining the relationship between condom use at first and last sexual experience among young people found that sexual activity with a boyfriend/girlfriend and sex without coercion was associated with condom use at both first and last sexual events. This study also found a significant association between school attendance and condom use at first and last sexual experience for females [35].

The lifetime number of sexual partners was strongly associated with HIV infection in both men and women. Women showed an increased odds of HIV infection with each additional partner over one while men did not indicate an increased odds until four or more sexual partners were reported. Men who reported having five or more sexual partners had the highest odds of HIV infection. Women who reported having up to four sexual partners had the highest odds of infection. Understanding the cultural factors that influence partnerships of sub-Saharan African countries, such as Lesotho, may identify the reason for an increased odds of HIV for women with only the addition of one partner while the same is not seen for men. Prior studies have reported that majority of women are unable to negotiate consistent male or female condom use [36]. It is possible that interventions that provide increased access to condoms may not be beneficial if women are unable to get their male partner to agree. Alternatively, studies have reported that improving women’s sexual autonomy and self-efficacy through training in use of female-initiated contraceptive and HIV/STI prevention gives them greater control and bargaining power within their sexual relationships [37].

Area of residence was associated with an increased risk of HIV-infection among both men and women while ecological zone was significant among only women. Living in a rural setting acted as protective factor where both men and women had significantly lower odds of being infected with HIV compared to those who lived in an urban setting. Women were significantly affected by both area of residence and ecological zone in which women who lived in the foothills or mountain region were at a lower risk of being HIV positive compared to women who lived in the lowlands. The increased odds of women living in the lowlands, which includes a large portion of urban areas, indicates a geographic characteristic that may be important to consider in public health programing. According to LePHIA data, in Lesotho’s the vast majority, 69.3%, of the population lived in the lowlands [16]. Ever living outside of Lesotho also had an association with HIV infection among women who have lived outside of Lesotho one to two times. Lesotho has a high rate of both internal and external migration and given its landlocked status, the country should consider working with surrounding areas in South Africa to develop HIV prevention messages and ART delivery programs for the Basotho population living temporarily in South Africa.

Wealth quintile was significantly associated with HIV infection among women. For every increase in unit of wealth, a women’s odds of being HIV positive decreased significantly. However, an increased level of wealth may not be obtainable by majority of the population as 49.7% of the country is living in poverty [38]. The large percentage of people living in poverty and the decreased odds of HIV infection with every increase in wealth quintile for women indicates a need for interventions and resources that provide relief to low income individuals, especially women. Structural interventions that focus on economic empowerment show promise in decreasing risk with cash transfer programs among young women in Malawi reducing the risk of HIV and HSV-2 infection [20]. It is important to note that our results did not find a significant association between increased level of wealth and HIV status. Until recently, multiple studies had found a positive association between wealth level and HIV status. Our results support the recent Andrus study findings that the positive association between HIV and wealth has weakened over time [39].

Medical circumcision was associated with less HIV infection among men. Men who had been medically circumcised were significantly less likely to be HIV positive compared to men who were not circumcised or received a traditional circumcision. The prevalence of voluntary male medical circumcision (VMMC) in the community is important as it reduces the risk of female-to-male transmission of HIV by 50–60% and is recognized as an cost-effective and impactful HIV intervention [40]. Through biological mechanisms, male circumcision has shown potential in decreasing a female partner’s risk of developing STI’s which can act as co-factor for HIV transmission [41]. According to LePHIA data, 36% of men reported haven been medically circumcised with the highest rates of medical circumcision among single men [16]. The significance of medical circumcision and the low rates seen in Lesotho indicates a need for interventions that focus on increasing awareness and accessibility of the procedure for young men and boys. However, the current high prevalence of traditional circumcision among men should be addressed during interventions as traditional circumcision does not protect from HIV transmission [10]] and is not always carried out by trained health professionals [42].

Despite prior efforts to increase male-friendly HIV-related services including testing, HIV status awareness was especially low among men at 71% nationally. Among men aged 35–49 years, awareness ranged from 67–85% with 5%–10% reporting being aware of their status, but were not receiving ART; this is the population with the highest male incidence at 3.01% per year as well as the age band (40–44 years) in which HIV prevalence peaks. This may be related to stigma or differences in health seeking behaviors among males. The low rate of awareness among men in Lesotho represents a significant opportunity for HIV transmission from older men to younger women and an area that should be a priority for prevention, testing, and treatment programs.

Among women aged 30–34 years, 12% reported being aware of their HIV-positive status but were not yet receiving ART [16]; HIV prevalence among this age group is 41%. Although Test and Start has increased the number of people on treatment, LePHIA data showed that not all persons who were aware of their status were receiving treatment. Testing activities should place specific emphasis on the importance of beginning treatment as soon as possible after an HIV-positive diagnosis given the percentage of persons, especially young persons, aware of their status but not yet receiving ART. Strengthening the linkage to care process in Test and Start among those testing positive could help reduce onward transmission.

The overall discordance of 16.6% (19.1% among men and 14.0% among women) between self-reported HIV status and ART use and ARV metabolite confirmed status may be indicative of stigma and feelings of an inability to disclose one’s status, despite Lesotho having one of the worst HIV epidemics in the world. Our analysis showed that disclosure among men is impacted by community and individual stigma. Among persons with metabolite confirmed ARVs, men were more likely to self-report both an HIV-negative status as well no ART use among those who self-reported a prior HIV-positive diagnosis. Women were more likely to over report being on ART. Over-reporting by women may be a side effect of greater health care utilization and access to ART message at clinics. Nonadherence to ARVs and/or disengagement from care threatens to reverse progress made towards 90-90-90 targets and viral suppression in Lesotho. Educational messages should be included with follow up appointments and ART pickups to reinforce the importance of adherence in achieving non-detectable viral loads and preventing HIV transmission. Stigma prevention messages should continue to be population focused, but ensure greater reach among men.

The discrepancy between self-disclosure of ART use and laboratory confirmed ART use is not limited to Lesotho. In South Africa, over one in three individuals, ages 40+, taking part in a home-based interview in a rural area had detectable ARVs in their blood, despite denying use of ARTs during the interview [28]. In a national survey in Kenya, ARV metabolites were present amongst 21% of respondents who reported an HIV-negative status [18]. Under-reporting ART use or an HIV-positive status can have negative implications for determining prevalence and ART coverage for countries and may reflect delayed progress towards the 90-90-90 targets. In future rounds of PHIA surveys, it may be worthwhile to look at using self-administered questionnaires or computer-assisted self-interviews, at least in part for sensitive questions.

Limitations of the current study include those common to studies relying on self-reported behavioral data. Social desirability bias may have influenced some participants to underreport the number of sexual partners they had in the last 12 months. The survey also asked questions about prior sexual behavior such as age at first sex, which may be subject to recall bias. Finally, because the survey was household based, persons who did not reside in the house the night before or who were away from home at the time of the survey were not included, which may affect migration-related data.

Throughout southern Africa, the substantial increase in ART coverage has resulted in decreased HIV incidence and mortality [29]. Although Lesotho is small, the epidemic is not geographically homogenous, with prevalence ranging from 17.8% to 29.3% across districts with statistically significant differences between men and women in eight of ten districts. As the country moves closer to achieving the 90-90-90 goals, it will be necessary to focus on these geographic and sex differences. Age was a significant predictor of HIV incidence, prevalence, and awareness. HIV education and prevention programs should focus heavily on younger age groups prior to and soon after sexual debut to prevent HIV transmission.

The findings of the survey showed significant room for improvement in increasing awareness of HIV status and reinforcing the need for continued HIV prevention and treatment efforts in Lesotho to prevent new infections. LePHIA data indicate that women are being infected at younger ages (15–24 years) and men at older ages (35–49 years), while prevalence increases rapidly until age 35–39 years for women and 40–44 years for men before decreasing again. While the survey was not adequately powered to provide incidence by age band, it seems that older men are infecting younger women [17]. Lesotho may face challenges in fully achieving epidemic control without an increased focus on preventing new infections, especially among AGYW and men 35–49 along with increased efforts to improve awareness of HIV status. Continued emphasis on understanding risk factors for new infections among men and young women, including age-disparate relationships, will lead to better-targeted testing programs, such as index case testing, self-testing, and HIV recency surveillance, and interventions to increase awareness and reduce incidence among these populations.
